# A statistical and numerical modeling approach for spatiotemporal reconstruction of glaciations in the Central Asian mountains

**DOI:** 10.1016/j.mex.2020.100820

**Published:** 2020-02-21

**Authors:** Sourav Saha, Lewis A. Owen, Elizabeth N. Orr, Marc W. Caffee

**Affiliations:** a Department of Earth, Planetary and Space Sciences, University of California, Los Angeles, CA 90095, USA; b Department of Marine, Earth, and Atmospheric Sciences, North Carolina State University, Raleigh, NC 27695, USA; c German Research Centre for Geosciences (GFZ), University of Potsdam, Institute for Geosciences, 14476 Potsdam, Germany; d Department of Physics, Department of Earth, Atmospheric and Planetary Sciences, Purdue University, West Lafayette, IN 47907, USA

**Keywords:** Glaciation, Cosmogenic isotopes, Paleoclimate modeling, Cluster analysis, Principal component analysis, Equilibrium-line altitudes

## Abstract

Reconstructing Quaternary regional glaciations throughout the Himalaya, Tibet, and the adjoining mountains in Central Asia is challenging due to geological biases towards limited preservation of glacial deposits and chronological uncertainties. Here, we offer several statistical and mathematical model codes in R, in excel, and in MATLAB useful to develop regional glacial chronostratigraphies, especially in areas with distinct orographically-modulated climate. A complete R code is provided to generate a regional climate map using Cluster Analysis (CA) and Principal Component Analysis (PCA). Additional R codes include reduced chi-squared, Chauvenet's criterion, radial plotter/abanico plot, finite mixture model, and Student's *t*-test. These methods are useful in reconstructing the timing of local and regional glacial chronologies. An excel code to calculate equilibrium-line altitudes (ELAs) and steps to reconstruct glacier hypsometry are also made available to further aid to our understanding of the extent of paleoglaciations. A MATLAB code of the linear glacier flow model is included to reconstruct paleotemperatures using the length and slope of a glacier during past advances.

• R statistical codes can be used/modified without restrictions for other researchers.
• Easy steps to calculate ELAs and glacier hypsometry from the same data.
• Paleo-temperature reconstruction utilizes already developed glacial chronologies and maps.

## Graphical abstract

See ref. [1].

**Figure:**
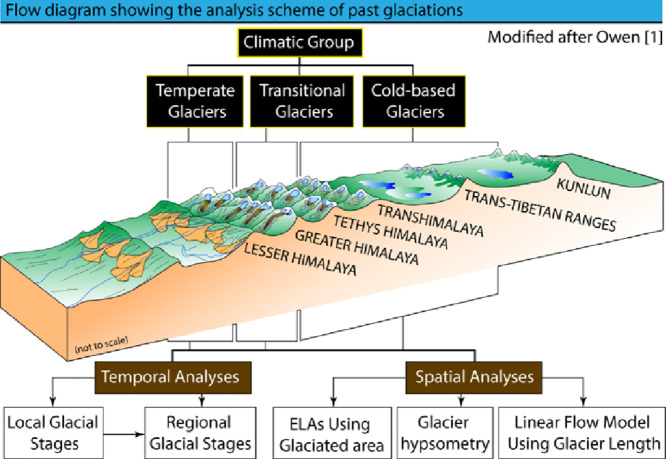

**Table:** Specification table

Subject Area:	*Earth and Planetary Sciences*
More specific subject area:	*Glacial Geology; Geomorphology; Paleoclimatology*
Method name:	***Climate statistics–****Cluster Analysis, Principal Component Analysis, Pearson's product-moment correlation, analysis of similarities.* ***Age statistics–****Reduced Chi-squared (χ^2^) test, Chauvenet's criterion, arithmetic and weighted mean, median, finite mixture model, radial plotter, Fisher's f-test, Student's t-test.* ***Equilibrium-line altitudes (ELAs)****– area-altitude (AA), area accumulation ratio (AAR), toe-headwall accumulation ratio (THAR).* ***Glacier hypsometry****and****Linear inverse glacier flow model***
Name and reference of the original method:	***Climate statistics–*** 1 *Clarke, K.R., 1993. Non-parametric multivariate analysis of changes in community structure. Australian Journal of Ecology 18, 117–143.* 2 *Romesburg, C., 2004. Cluster Analysis for Researchers. North Carolina, p. 330.* 3 *Sagredo, E. A., Lowell, T. V., 2012. Climatology of Andean glaciers: A framework to understand glacier response to climate change. Glob. Planet. Change 86–87, 101–109.* 4 *Seaby, R., Henderson, P., 2014. Community Analysis Package 5.0: Searching for structure in community data. PISCES Conserv. Ltd., Engl., p. 186.* 5 *Zuming, L. A. I., Maohuan, H., 1989. A numerical classification of glaciers in China by means of glaciological indices at the equilibrium line. Snow Cover Glacier Var. (Proceedings Balt. Symp. Maryland), 103–111.* ***Age statistics–*** 6 *Applegate, P. J., Urban, N. M., Keller, K., Lowell, T. V., Laabs, B. J. C., Kelly, M. A., Alley, R. B., 2012. Improved moraine age interpretations through explicit matching of geomorphic process models to cosmogenic nuclide measurements from single landforms. Quat. Res. 77, 293–304.* 7 *Galbraith, R., Roberts, R., Laslett, G., Yoshida, H., Olley, J., 1999. Optical Dating of Single and Multiple Grains of Quartz from Jinmium Rock Shelter, Northern Australia: Part I, Experimental Design and Statistical Models. Archaeometry 41, 339–364.* 8 *Taylor, J. R., 1997. An Introduction to Error Analysis. Second Edition, University Science Books, Sausalito, Calif.* ***Morphometric models–*** 9 *Oerlemans, J., 2005. Extracting a climate signal from 169 glacier records. Science 308, 675–677.* 10 *Osmaston, H., 2005. Estimates of glacier equilibrium line altitudes by the Area x Altitude, the Area x Altitude Balance Ratio and the Area x Altitude Balance Index methods and their validation. Quat. Int. 138–139, 22–31.* 11 *Pike, R.J., and Wilson, S.E., 1971. Elevation-relief ratio, hypsometric integral, and geomorphic area-altitude analysis. Geological Society of America Bulletin, 82(4), pp.1079–1084.*
Resource availability:	https://expage.github.io/data.html https://cran.r-project.org https://github.com/vegandevs/vegan https://cran.r-project.org/web/packages/Luminescence/index.html http://www.geog.ucsb.edu/~bodo/TRMM/ https://crudata.uea.ac.uk/cru/data/hrg/tmc/ https://www.glims.org/RGI/rgi60_dl.html https://search.earthdata.nasa.gov/search https://www.osc.edu/

## Method details

**Fig. 1:**
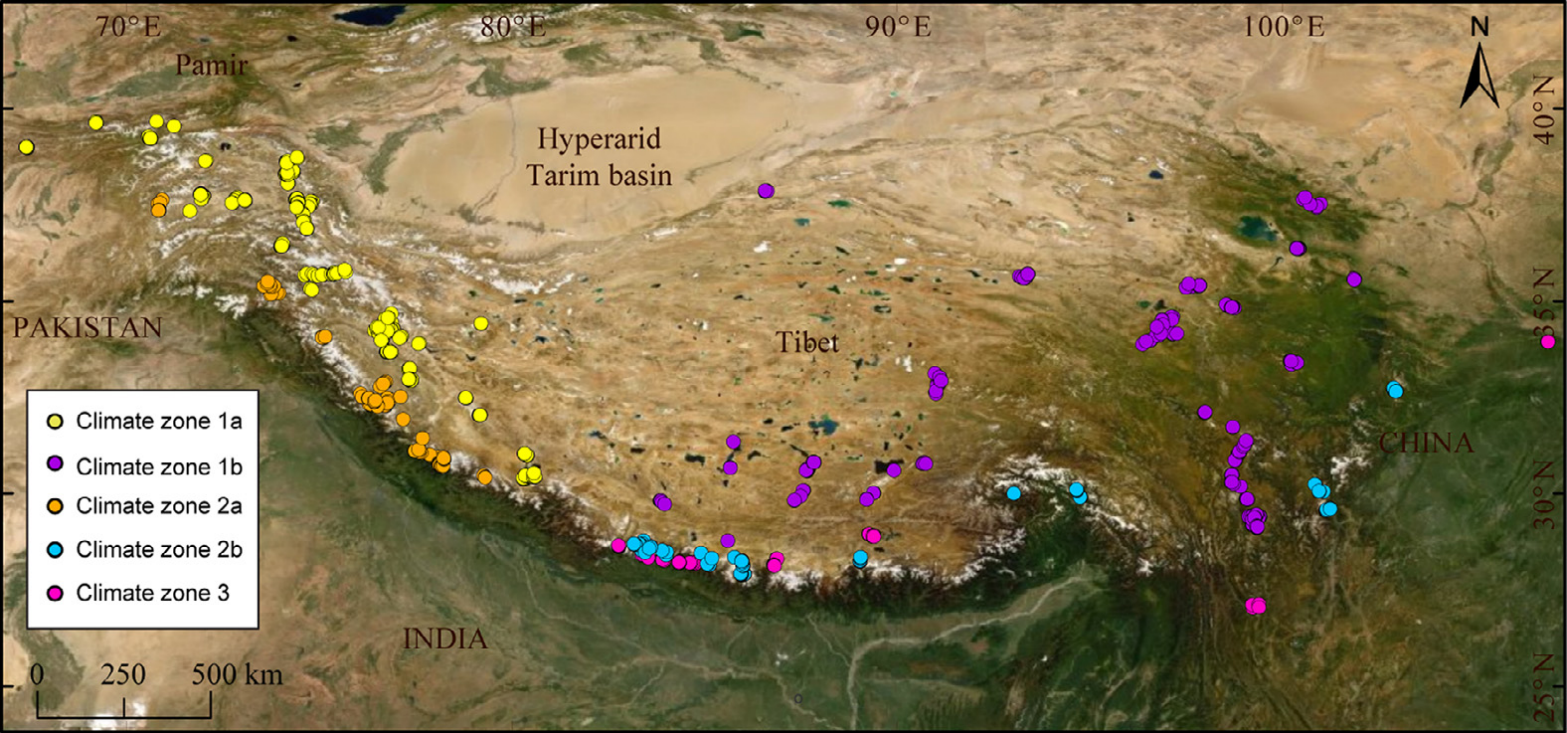
Hillshade map showing the location of ^10^Be and ^26^Al dated glaciated valleys across the Himalayan-Tibetan orogen. ^10^Be and ^26^Al dates of each studied valleys are color-coded based on the climatic zone map shown in Fig. 3B. See Saha et al. [3] for more details. Age data were compiled from http://expage.github.io/data.html.

With the increased chronological reconstructions over the past two decades throughout the glaciated mountains of Central Asia (Fig. 1), it is now possible to use different statistical and numerical models effectively to reconstruct regional glacial stages [2,^3^,4]. Here, we offer R, Excel, and MATLAB codes of several visual, statistical, and numerical models that include Cluster Analysis (CA), Principal Component Analysis (PCA), reduced chi-squared test (χ^2^), Chauvenet's criterion, radial plotter, abanico plot, finite mixture model, Student's *t*-test, equilibrium-line altitudes (ELAs) and glacier hypsometry, and linear (inverse) glacier flow model. These methods may be broadly categorized into climate statistics (e.g., CA, PCA), age statistics (e.g., χ^2^, Chauvenet's criterion, radial plotter, abanico plot, finite mixture model, *t*-test), and morphometric models (e.g., ELAs, hypsometry, flow model). Our objectives are (i) to identify climatically distinct groups of glaciers that are modulated by topography and orography; and (ii) to develop robust regional glacial stages in each climatic region for spatiotemporal comparison. The codes presented here are straightforward and can be easily modified or adapted to a more complex computational environment and a wide variety of geological settings. These codes are also powerful to help extract both temporal and spatial aspects of paleoglaciations, offering critical insight into the regional pattern of paleoglaciations.

We aim that these methods and codes would help quantify chronological data, especially local and regional moraine ages, in a much easier and robust way to the new user. All the necessary data and templates are provided in the supplementary materials. The software programs used to generate the codes are either publicly available (e.g., R and R packages) or readily purchasable (e.g., Microsoft Excel, MATLAB). The only exception is the Read ArcGrid program, which could be obtained by request since it is not proprietary to this study.

## Climatic statistics

### Data extraction

**Fig. 2:**
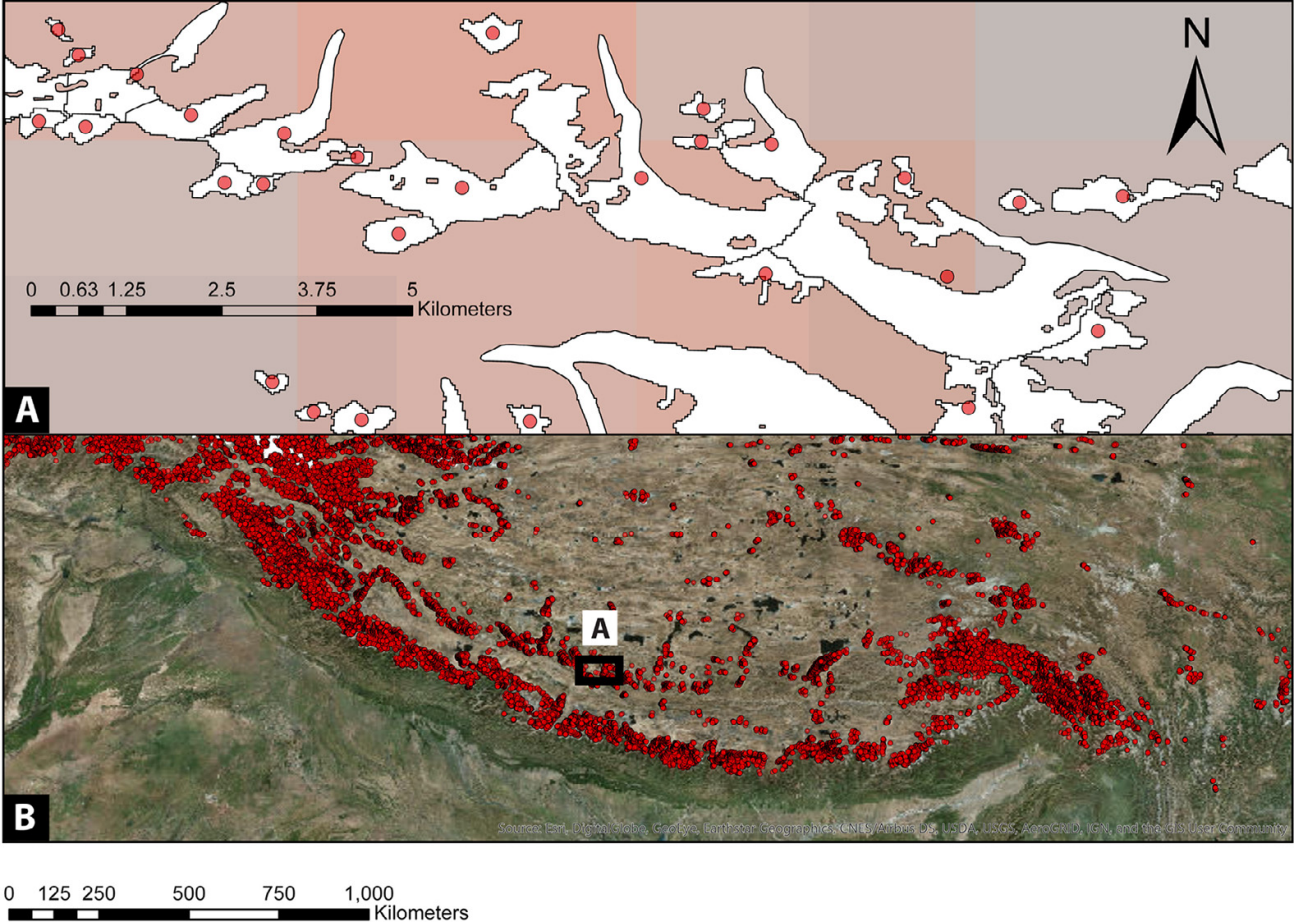
(A) Glacier polygons, derived from the Randolph Glacier Inventory (RGI 6.0), were converted into points using their central location. (B) Each point (glacier) was then used to extract climate data. The glaciers are superimposed on the TRMM derived gridded precipitation datasets (A). The point cloud in the lower panel (B) is superimposed on Google Earth images.

Both CRU CL 2.0 (10′ latitude/longitude) reanalysis temperature (*T* in °*C*) data (https://crudata.uea.ac.uk/cru/data/hrg/tmc/) and TRMM derived precipitation (*PCP in mm*) data (4-km-horizontal x 250-m-vertical; *http://www.geog.ucsb.edu/~bodo/TRMM/*) are first rescaled to 18 × 18 km grid cell for each month in ArcGIS 10.5; averaged over 30 and 12 years, respectively. CRU CL 2.0 precipitation reanalysis data were not used due to high uncertainties for high-altitude places [5]. Approximately 42,511 glacier polygons (vector samples), derived from the Randolph Glacier Inventory (RGI 6.0), are then converted into points using the *Feature to Point* tool in ArcGIS 10.5 (Fig. 2). Subsequently, climate data are extracted for each point (glacier/sample) from the gridded datasets using the *Extract Multi Values to Points* tool (Fig. 2). Nine climate parameters are derived from the primary data for each glacier (Supplementary Table S1). These are:

1 Annual mean temperature (*T*(*x*) = $\sum_{x=1}^{12}\frac{T(x)}{12})$(i)
2 Annual temperature range (*T*(*x*) = *T*(*x*) max*.–T*(*x*) min*.*)(ii)
3 Annual total precipitation (*PCP^a^*(*x*) = $\sum_{x=1}^{12}PCP\left(x\right))$(iii)
4 Annual precipitation range (*PCP^r^*(*x*) = *PCP*(*x*) max*.–PCP*(*x*) min*.*)(iv)
5 Total summer (monsoon) pecipitation (*PCP^s^*(*x*) = $\sum_{x=1}^{4}PCP\left(x\right))$ for *JJAS* (v)
6 Total winter (westerlies) precipitation (*PCP^w^*(*x*) = $\sum_{x=1}^{4}PCP\left(x\right))$ for *DJFM* (vi)
7 Seasonality Concentration Index ($SCI=\frac{\sqrt{\frac{1}{12-1}\sum_{x=1}^{12}{\left(PCP(x)-\text{PCP}\right)}^{2}}}{PCP}$) (vii)

where PCP is mean annual precipitation [5]

8 Relative Entropy $(RE=\sum_{m=1}^{12}\;PCP\;m\left(x\right)\;log$*_2_* (12 *PCP* *m* (*x*))) (viii)

where *m* is a month and $PCP\;m\left(x\right)=\frac{PCP(x)}{\text{PCP}}$ [6] and

9 Dimensionless Seasonality Index ($DSI=RE\frac{\text{PCP}}{R}$) (ix)

Where ***R*** is a constant scaling factor introduced to make precipitation dimensionless. We choose ***R*** as the maximum of ($\sum_{x=1}^{12}PCP\left(x\right))\;$among all sample glaciers (see supplementary table S1).

*SCI* indicates relative precipitation variability in each month compared to the mean annual [6]. *RE* is likely associated with the number of ‘wet’ months and reaches its maximum value of log*_2_ 12* when annual precipitation is concentrated in one single month [7]. According to definition (ix), *DSI* is zero when either PCP (completely dry location) or *RE* (PCP distributed uniformly throughout the year) are zero and maximum (*log_2_ 12*) when ***R*** is concentrated in a single month [7]. Due to the nature, units, and dissimilar ranges of the climate parameters, all data, except *DSI*, are scaled to lie between 0 and 1, by diving by the maximum value in the sample population for respective parameters (e.g., *T*(*x*)*/T*(*x*) max*.*); Supplementary Table S1).

### Statistical model

**Fig. 3:**
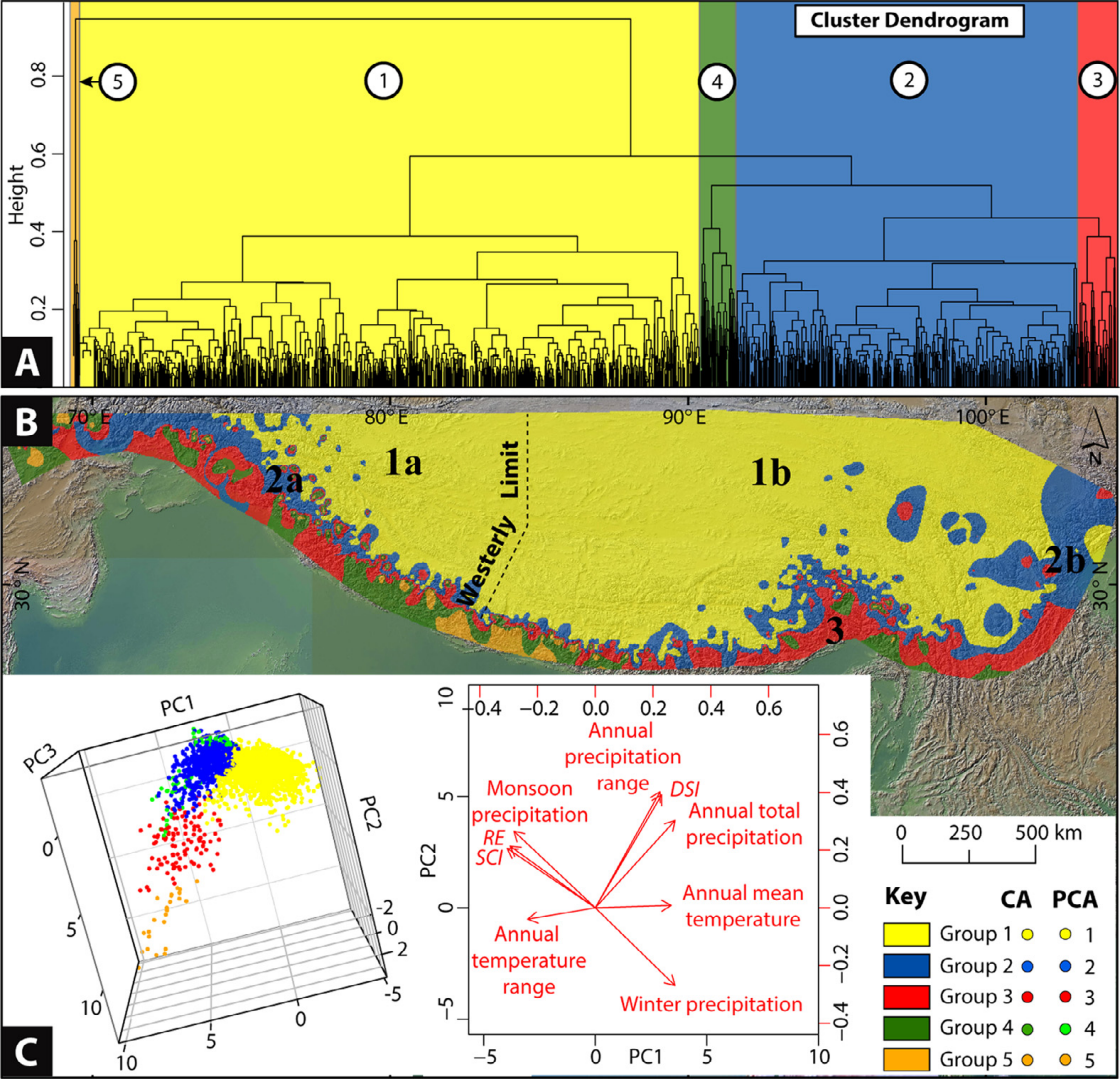
Distinct climate groups are identified using Cluster (CA) and Principal Component Analyses (PCA) for Himalayan-Tibetan glaciers. (A) The cluster dendrogram shows groups of most identical glaciers with similar climatic characteristics (see Section 1.1). Each terminal of the hierarchical tree represents a single glacier, and they are joined with each other based on a certain level of similarity; most similar glaciers are clustered together at each hierarchy. Five (climate) groups of glaciers are first identified using CA. (B) A climate zone map (superimposed over a hillshade map) is generated using the interpolation method and using each glacier's CA grouping. The dotted line represents the mid-latitudinal westerlies limit (from the west). Further subdivisions (defined as ‘Climatic zones’) of glaciers based on the meridional limit of westerlies are proposed in Saha et al. [3]. For example, group 1 is subdivided into climatic zone 1a (i.e., Arid and semiarid Transhimalaya, northwest Tibet, Pamir, and the Tian Shan) and zone 1b (i.e., Arid and semiarid southern and northeastern Tibet). Group 4 and 5, as identified in the CA dendrogram, are discarded from further analysis since they only represent end members in PCA analysis (C). Due to no ^10^Be and ^26^Al dates in group 3, no subdivisions are made. See Saha et al. [3] for a detail explanation of the scheme. The color-coding of circles in Fig. 1 is based on this subdivision (climatic zones). (C) 3D plot showing the corresponding PCA results for PC1, PC2, and PC3 and the biplot showing the magnitude and sign of each parameter's contribution to the PC1 and PC2. Both methods indicate identical grouping. The figure is modified after Saha et al. [3].

We performed CA [8] using all nine climate parameters extracted using glaciers as points (Supplementary Table S1). CA is often favored to classify samples (e.g., glaciers) based on the degree of similarity among them given a defined set of parameters [9,8]. Euclidean (linear) distances between any pair of glaciers in a multidimensional (in our case no. of dimensions are nine) Cartesian space is calculated in R [10]. Using the unweighted pair-group method with arithmetic averaging (UPGMA; [11]), finally, a cluster dendrogram is produced by grouping together the most similar (i.e., least linear distances) sets of glaciers (Fig. 3A). In addition, the significance of our clusters is tested using Pearson's product-moment correlation between the sample Euclidean and cophenetic distances [12]. A higher positive correlation is expected for our CA groupings to be validated [9]. We also performed analysis of similarities (ANOSIM; [13]) between sample Euclidean distances and CA defined groups (with 600 permutations) to calculate *R*. Where the value of *R* (constrained between –1 to 1) is more positive, the samples within groups are more similar than would be expected by random chance [9] (Fig. 3B). A similar statistical model was successfully applied to Andean glaciers by Sagredo and Lowell [9]. In Saha et al. [3], we conclude that this type of model equally works best for Himalayan-Tibetan glaciers and is therefore useful in comparison to the existing manual delineation based on arbitrary topographic ranges [2, 14].

Following is the R code for CA. The Excel sheet in the supplementary Table S1 titled “For_R” can be used as a template; the current R code is designed to read a comma-delimited (*csv) file format. Note that the analyzed climate data provided in S1 is dense, and hence, the computation is performed at the Ohio Supercomputer Center ([15]; https://www.osc.edu/). Readers who intend to use/modify the code must reduce the data volume in Table S1 (sheet For_R) to try in the general computer system.

# Cluster (CA) hierarchical model

#——————————————————————-

# Setting up the directory and file input

## "Directory need to change if using in other computer"

setwd("C:/users/folder_name/")

# file input

clima <- read.csv(file.choose()) # read the csv file

head(clima) # for quick look of the file to see the variable names

# Notes: the variables must be transformed into dimensionless values

# using a maximum of each climatic variables; values range from 0 to 1

# Note: Log transformation can be used if the data is skewed; here no log transformation is attempted since that negatively affect thegrouping

#——————————————————————-

# Check the installation of these necessary packages

#install package "vegan"

install.packages("vegan")

library(vegan) #load vegan package

#install package "rgl"

install.packages("rgl")

library(rgl) #load rgl 3d package

#——————————————————————-

# treating as data frame

pre <- (clima[ , c(1, 11)]) # Annual total precipitation

temp <- (clima[ , c(1, 9)]) # Annual mean temperature

rangeT <- (clima[ , c(1, 10)]) # Annual temperature range

rangeP <- (clima[ , c(1, 12)]) # Annual precipitation range

monP <- (clima[, c(1,13)]) # Summer monsoon precipitation (J,J,A,S,O)

wintP <- (clima[, c(1,14)]) # Winter and late winter precipitation (D,J,F,M,A,M)

# Other indices

SCI <- (clima[ , c(1, 15)]) # SCI= stdv of 12/ave of 12

RE <- (clima[ , c(1, 16)]) # relative entropy [Pascale et al., 2015]

DSI <- (clima[ , c(1, 17)]) # dimensionless seasonality index [Pascale et al., 2015]

#——————————————————————-

# Converting the data frame into a matrix called 'data'

data<-cbind(pre[,2],temp[,2],rangeT[,2],rangeP[,2],monP[,2],wintP[,2],SCI[,2],RE[,2],DSI[,2])

#——————————————————————-

# Cluster Analysis modified after Zuming and Maohuan (1989)

# To create a cluster dendrogram of each individual variable

# To create a distance matrix using Euclidean distance method, i.e., measuring distance between scatter points (method=Euclidean)

clima.dist <- vegdist(data,method="euclidean")

# Uses UPGMA on distance matrix to create hierarchical structure

clima.upgma <- hclust(clima.dist, method = "average" )

#———————————————————–

# Plotting and grouping the data

# main cluster dendrogram using UPGMA (Romesburg, 2004)

plot(clima.upgma, hang = -1)

# Grouping/clustering

groups <- cutree(clima.upgma, *k* = 2:15) # cut tree into *k*=no of groups/clusters; here 2 groups to 15 groups are chosen

# draw dendrogram with red borders around the group clusters

rect.hclust(clima.upgma, *k* = 5, border="red") # here only five groups are chosen to highlight between 2 and 15

#——————————————————————-

# To print the group table in workspace

print.table(groups)

# combined all the data that needs to print a text file

CAtable= cbind(groups, clima[, 1:17])

# To create the final text file

## "Directory need to change if using in another computer"

write.table(CAtable, "C:/users/folder_names/CA_groups.txt", sep="\t")

#——————————————————————-

# 1st significance test of the clustering

# Calculate the cophenetic distances (Seaby and Henderson, 2014)

clima.coph.dist <- cophenetic(clima.upgma)

#check correlation between clima.dist and clima.upgma (it gives you a r^2 value)

cor.test(clima.dist, cophenetic(clima.upgma))

#——————————————————————-

# 2nd significance test of the clustering: “ANOSIM”

# Read the table generated in the previous steps

climaCA <- read.table("CA_groups.txt", sep="\t")

# Significance test using ANOSIM (Clarke, 1993)

# from a group of 2 to 15; you can choose any one group to test

# GROUP of 2

# anosim(clima.dist, climaCA [,1], permutations = 999)

# GROUP of 3

# anosim(clima.dist, climaCA [,2], permutations = 999)

# GROUP of 4

# anosim(clima.dist, climaCA [,3], permutations = 999)

# GROUP of 5

anosim(clima.dist, climaCA [,4], permutations = 999) # no. of permutations can be reduced to fasten the process

# GROUP of 6

# anosim(clima.dist, climaCA [,5], permutations = 999)

# GROUP of 7

# anosim(clima.dist, climaCA [,6], permutations = 999)

# GROUP of 8

# anosim(clima.dist, climaCA [,7], permutations = 999)

# GROUP of 9

# anosim(clima.dist, climaCA [,8], permutations = 999)

# GROUP of 10

# anosim(clima.dist, climaCA [,9], permutations = 999)

# GROUP of 11

# anosim(clima.dist, climaCA [,10], permutations = 999)

# GROUP of 12

# anosim(clima.dist, climaCA [,11], permutations = 999)

# GROUP of 13

anosim(clima.dist, climaCA [,12], permutations = 999)

# GROUP of 14

# anosim(clima.dist, climaCA [,13], permutations = 999)

# GROUP of 15

# anosim(clima.dist, climaCA [,14], permutations = 999)

# Copy and paste the results in a word file if required

#——————————————————————-

CA results obtained using the R code were further evaluated using PCA (Fig. 3C). Such a comparison using two different statistical methods is not uncommon and proved successful in other fields [16]. We performed PCA to correlate climate parameters and represent them as a smaller set of uncorrelated (orthogonal) variables called principal components (PCs; Fig. 3C) [9]. PCs geometrically capture the directions of maximum variation in the data [3] and therefore, are used to compare and refine our CA generated groups (Fig. 3C). For example, please see Fig. 6 in Saha et al. [3].

#——————————————————————-

# Continuing from the previous code

# Principal Component Analysis (PCA)

# Setting up the directory and file input

## "Directory need to change if using in another computer"

setwd("C:/users/folder_name/")

# Read the text file just generated file

climaPCA <- read.table("CA_groups.txt", sep="\t")

head(climaPCA) # for quick look

clima <- (cbind(climaPCA[, 23:31])) # binding the climate variables to a matrix

#——————————————————————-

# Initial plotting to see the relationship among variables

# Plot pairwise scatterplots

pairs(clima)

#——————————————————————-

# Giving new column names

colnames(clima) <- c("AMeanTemp","ATempRange","ATotPrec","APrecRange","MonPrec","WintPrec","SCI", "RE","DSI")

#——————————————————————-

# Computing the Principal Components (PC)

prin_comp <- prcomp (clima, scale. = *T*)

names(prin_comp)

"sdev" "rotation" "center" "x"

#outputs the mean variables

prin_comp$center

#outputs the standard deviation of variables

prin_comp$scale

#rotation measure provides the principal component loading

prin_comp$rotation

#Let's plot the resultant principal components

biplot(prin_comp, scale = 0)

#compute standard deviation of each principal component

std_dev <- prin_comp$sdev

std_dev

#compute variance

pr_var <- std_dev^2

pr_var

#proportion of variance explained

prop_varex <- pr_var/sum(pr_var)

prop_varex

#——————————————————————-

# summarise PCA results

summary(prin_comp) # The summary method describes the importance of PCs.

prin_comp $loadings # This is what you need to interpret

prin_comp $scores # Print the scores (projections)

# Save the txt file for PCA loadings

## "Directory need to change if using in other computer"

write.table(prin_comp$scores, "C:/users/folder_name/PCA$loadings.txt", sep="\t")

# Write the txt file for PCA scores

write.table(prin_comp$scores, "C:/users/folder_name/PCA$scores.txt", sep="\t")

#——————————————————————–

# Ploting first 3 PCA scores

pc1 <- prin_comp$scores [ , c(1)]

pc2 <- prin_comp$scores [ , c(2)]

pc3 <- prin_comp$scores [ , c(3)]

# Creating a matrix

PC <- cbind(climaPCA[, 1:14],pc1,pc2,pc3)

#——————————————————————–

# Colored presentation based on the number of groups

# To save time chose only the group that you would like to plot

# Other groups are provided for the reader's convenience (i.e., optional)

#——————————————————————–

# GROUP: 2

#——————————————————————–

#color coding groups of 2

colorgroups<-function(PC){

Group= vector(length=nrow(PC))

*x*<-cbind(PC,Group)

for(i in 1:nrow(x)){

# Number number of if statements = Number of groups

if(x[*i*,1]==1) {x[*i*,18]<-"yellow"}

if(x[*i*,1]==2) {x[*i*,18]<-"blue"}

}

print(x)

}

groupscolored <- colorgroups(PC)

# plot using any plot function, but for the argument "col=" use "col=groupscolored

# PC1 vs PC2

plot (groupscolored[,15],groupscolored[,16], col=groupscolored[,18],

main = "GROUP:2″, xlab="PC1", ylab="PC2",pch=16)

# PC1 vs PC3

plot (groupscolored[,15],groupscolored[,17], col=groupscolored[,18],

main = "GROUP:2″, xlab="PC1", ylab="PC3",pch=16)

# PC2 vs PC3

plot (groupscolored[,16],groupscolored[,17], col=groupscolored[,18],

main = "GROUP:2″, xlab="PC2", ylab="PC3",pch=16)

plot3d (groupscolored[,15],groupscolored[,16],groupscolored[,17], col=groupscolored[,18],

xlab="PC1", ylab="PC2", zlab="PC3", pch=16, size=8)

grid3d ("x", at = NULL, col = "gray", lwd = 1, lty = 1, *n* = 5)

grid3d ("*y*+", at = NULL, col = "gray", lwd = 1, lty = 1, *n* = 5)

grid3d ("z", at = NULL, col = "gray", lwd = 1, lty = 1, *n* = 5)

title3d("GROUP of 2″, col = 'red', line = 3)

# rgl snapshot and pdf vector

rgl.snapshot("GROUP_2.png", fmt="png")

rgl.postscript("GROUP_2.pdf", "pdf", drawText = TRUE)

#——————————————————————–

# GROUP: 3

#——————————————————————–

#color coding groups of 3

colorgroups<-function(PC){

Group= vector(length=nrow(PC))

*x*<-cbind(PC,Group)

for(i in 1:nrow(x)){

# Number of if statements = Number of groups

if(x[*i*,2]==1) {x[*i*,18]<-"yellow"}

if(x[*i*,2]==2) {x[*i*,18]<-"blue"}

if(x[*i*,2]==3) {x[*i*,18]<-"red"}

}

print(x)

}

groupscolored <- colorgroups(PC)

# plot using any plot function, but for the argument "col=" use "col=groupscolored

# PC1 vs PC2

plot (groupscolored[,15],groupscolored[,16], col=groupscolored[,18],

main = "GROUP: 3″, xlab="PC1", ylab="PC2",pch=16)

# PC1 vs PC3

plot (groupscolored[,15],groupscolored[,17], col=groupscolored[,18],

main = "GROUP: 3″, xlab="PC1", ylab="PC3",pch=16)

# PC2 vs PC3

plot (groupscolored[,16],groupscolored[,17], col=groupscolored[,18],

main = "GROUP: 3″, xlab="PC2", ylab="PC3",pch=16)

plot3d (groupscolored[,15],groupscolored[,16],groupscolored[,17], col=groupscolored[,18],

xlab="PC1", ylab="PC2", zlab="PC3", pch=16, size=8)

grid3d ("x", at = NULL, col = "gray", lwd = 1, lty = 1, *n* = 5)

grid3d ("*y*+", at = NULL, col = "gray", lwd = 1, lty = 1, *n* = 5)

grid3d ("z", at = NULL, col = "gray", lwd = 1, lty = 1, *n* = 5)

title3d("GROUP of 3″, col = 'red', line = 3)

# rgl snapshot and pdf vector

rgl.snapshot("GROUP_3.png", fmt="png")

rgl.postscript("GROUP_3.pdf", "pdf", drawText = TRUE)

#——————————————————————–

# GROUP: 4

#——————————————————————–

#color coding groups of 4

colorgroups<-function(PC){

Group= vector(length=nrow(PC))

*x*<-cbind(PC,Group)

for(i in 1:nrow(x)){

# Number of if statements = Number of groups

if(x[*i*,3]==1) {x[*i*,18]<-"yellow"}

if(x[*i*,3]==2) {x[*i*,18]<-"blue"}

if(x[*i*,3]==3) {x[*i*,18]<-"red"}

if(x[*i*,3]==4) {x[*i*,18]<-"green"}

}

print(x)

}

groupscolored <- colorgroups(PC)

# plot using any plot function, but for the argument "col=" use "col=groupscolored

# PC1 vs PC2

plot (groupscolored[,15],groupscolored[,16], col=groupscolored[,18],

main = "GROUP: 4″, xlab="PC1", ylab="PC2",pch=16)

# PC1 vs PC3

plot (groupscolored[,15],groupscolored[,17], col=groupscolored[,18],

main = "GROUP: 4″, xlab="PC1", ylab="PC3",pch=16)

# PC2 vs PC3

plot (groupscolored[,16],groupscolored[,17], col=groupscolored[,18],

main = "GROUP: 4″, xlab="PC2", ylab="PC3",pch=16)

plot3d (groupscolored[,15],groupscolored[,16],groupscolored[,17], col=groupscolored[,18],

xlab="PC1", ylab="PC2", zlab="PC3", pch=16, size=8)

grid3d ("x", at = NULL, col = "gray", lwd = 1, lty = 1, *n* = 5)

grid3d ("*y*+", at = NULL, col = "gray", lwd = 1, lty = 1, *n* = 5)

grid3d ("z", at = NULL, col = "gray", lwd = 1, lty = 1, *n* = 5)

title3d("GROUP of 4″, col = 'red', line = 3)

# rgl snapshot and pdf vector

rgl.snapshot("GROUP_4.png", fmt="png")

rgl.postscript("GROUP_4.pdf", "pdf", drawText = TRUE)

#——————————————————————–

**# GROUP: 5 (used in this study)**

#——————————————————————–

#color coding groups of 5

colorgroups<-function(PC){

Group= vector(length=nrow(PC))

*x*<-cbind(PC,Group)

for(i in 1:nrow(x)){

# Number of if statements = Number of groups

if(x[*i*,4]==1) {x[*i*,18]<-"yellow"}

if(x[*i*,4]==2) {x[*i*,18]<-"blue"}

if(x[*i*,4]==3) {x[*i*,18]<-"red"}

if(x[*i*,4]==4) {x[*i*,18]<-"green"}

if(x[*i*,4]==5) {x[*i*,18]<-"orange"}

}

print(x)

}

groupscolored <- colorgroups(PC)

# plot using any plot function, but for the argument "col=" use "col=groupscolored

# PC1 vs PC2

plot (groupscolored[,15],groupscolored[,16], col=groupscolored[,18],

main = "GROUP: 5″, xlab="PC1", ylab="PC2",pch=16)

# PC1 vs PC3

plot (groupscolored[,15],groupscolored[,17], col=groupscolored[,18],

main = "GROUP: 5″, xlab="PC1", ylab="PC3",pch=16)

# PC2 vs PC3

plot (groupscolored[,16],groupscolored[,17], col=groupscolored[,18],

main = "GROUP: 5″, xlab="PC2", ylab="PC3",pch=16)

plot3d (groupscolored[,15],groupscolored[,16],groupscolored[,17], col=groupscolored[,18],

xlab="PC1", ylab="PC2", zlab="PC3", pch=16, size=8)

grid3d ("x", at = NULL, col = "gray", lwd = 1, lty = 1, *n* = 5)

grid3d ("*y*+", at = NULL, col = "gray", lwd = 1, lty = 1, *n* = 5)

grid3d ("z", at = NULL, col = "gray", lwd = 1, lty = 1, *n* = 5)

title3d("GROUP of 5″, col = 'red', line = 3)

grid3d ("x", at = NULL, col = "gray", lwd = 1, lty = 1, *n* = 5)

grid3d ("*y*+", at = NULL, col = "gray", lwd = 1, lty = 1, *n* = 5)

grid3d ("z", at = NULL, col = "gray", lwd = 1, lty = 1, *n* = 5)

title3d("GROUP of 5″, col = 'red', line = 3)

# rgl snapshot and pdf vector

rgl.snapshot("GROUP_5.png", fmt="png")

rgl.postscript("GROUP_5.pdf", "pdf", drawText = TRUE)

#——————————————————————–

# GROUP: 6

#——————————————————————–

#color coding groups of 6

colorgroups<-function(PC){

Group= vector(length=nrow(PC))

*x*<-cbind(PC,Group)

for(i in 1:nrow(x)){

# Number of if statements = Number of groups

if(x[*i*,5]==1) {x[*i*,18]<-"yellow"}

if(x[*i*,5]==2) {x[*i*,18]<-"blue"}

if(x[*i*,5]==3) {x[*i*,18]<-"red"}

if(x[*i*,5]==4) {x[*i*,18]<-"green"}

if(x[*i*,5]==5) {x[*i*,18]<-"orange"}

if(x[*i*,5]==6) {x[*i*,18]<-"purple"}

}

print(x)

}

groupscolored <- colorgroups(PC)

# plot using any plot function, but for the argument "col=" use "col=groupscolored

# PC1 vs PC2

plot (groupscolored[,15],groupscolored[,16], col=groupscolored[,18],

main = "GROUP: 6″, xlab="PC1", ylab="PC2",pch=16)

# PC1 vs PC3

plot (groupscolored[,15],groupscolored[,17], col=groupscolored[,18],

main = "GROUP: 6″, xlab="PC1", ylab="PC3",pch=16)

# PC2 vs PC3

plot (groupscolored[,16],groupscolored[,17], col=groupscolored[,18],

main = "GROUP: 6″, xlab="PC2", ylab="PC3",pch=16)

plot3d (groupscolored[,15],groupscolored[,16],groupscolored[,17], col=groupscolored[,18],

xlab="PC1", ylab="PC2", zlab="PC3", pch=16, size=8)

grid3d ("x", at = NULL, col = "gray", lwd = 1, lty = 1, *n* = 5)

grid3d ("*y*+", at = NULL, col = "gray", lwd = 1, lty = 1, *n* = 5)

grid3d ("z", at = NULL, col = "gray", lwd = 1, lty = 1, *n* = 5)

title3d("GROUP of 6″, col = 'red', line = 3)

# rgl snapshot and pdf vector

rgl.snapshot("GROUP_6.png", fmt="png")

rgl.postscript("GROUP_6.pdf", "pdf", drawText = TRUE)

#——————————————————————–

# GROUP: 7

#——————————————————————–

#color coding groups of 7

colorgroups<-function(PC){

Group= vector(length=nrow(PC))

*x*<-cbind(PC,Group)

for(i in 1:nrow(x)){

# Number of if statements = Number of groups

if(x[*i*,6]==1) {x[*i*,18]<-"yellow"}

if(x[*i*,6]==2) {x[*i*,18]<-"blue"}

if(x[*i*,6]==3) {x[*i*,18]<-"red"}

if(x[*i*,6]==4) {x[*i*,18]<-"green"}

if(x[*i*,6]==5) {x[*i*,18]<-"orange"}

if(x[*i*,6]==6) {x[*i*,18]<-"purple"}

if(x[*i*,6]==7) {x[*i*,18]<-"deeppink"}

}

print(x)

}

groupscolored <- colorgroups(PC)

# plot using any plot function, but for the argument "col=" use "col=groupscolored

# PC1 vs PC2

plot (groupscolored[,15],groupscolored[,16], col=groupscolored[,18],

main = "GROUP: 7″, xlab="PC1", ylab="PC2",pch=16)

# PC1 vs PC3

plot (groupscolored[,15],groupscolored[,17], col=groupscolored[,18],

main = "GROUP: 7″, xlab="PC1", ylab="PC3",pch=16)

# PC2 vs PC3

plot (groupscolored[,16],groupscolored[,17], col=groupscolored[,18],

main = "GROUP: 7″, xlab="PC2", ylab="PC3",pch=16)

plot3d (groupscolored[,15],groupscolored[,16],groupscolored[,17], col=groupscolored[,18],

xlab="PC1", ylab="PC2", zlab="PC3", pch=16, size=8)

grid3d ("x", at = NULL, col = "gray", lwd = 1, lty = 1, *n* = 5)

grid3d ("*y*+", at = NULL, col = "gray", lwd = 1, lty = 1, *n* = 5)

grid3d ("z", at = NULL, col = "gray", lwd = 1, lty = 1, *n* = 5)

title3d("GROUP of 7″, col = 'red', line = 3)

# rgl snapshot and pdf vector

rgl.snapshot("GROUP_7.png", fmt="png")

rgl.postscript("GROUP_7.pdf", "pdf", drawText = TRUE)

#——————————————————————–

# GROUP: 8

#——————————————————————–

#color coding groups of 8

colorgroups<-function(PC){

Group= vector(length=nrow(PC))

*x*<-cbind(PC,Group)

for(i in 1:nrow(x)){

# Number of if statements = Number of groups

if(x[*i*,7]==1) {x[*i*,18]<-"yellow"}

if(x[*i*,7]==2) {x[*i*,18]<-"blue"}

if(x[*i*,7]==3) {x[*i*,18]<-"red"}

if(x[*i*,7]==4) {x[*i*,18]<-"green"}

if(x[*i*,7]==5) {x[*i*,18]<-"orange"}

if(x[*i*,7]==6) {x[*i*,18]<-"purple"}

if(x[*i*,7]==7) {x[*i*,18]<-"deeppink"}

if(x[*i*,7]==8) {x[*i*,18]<-"chocolate"}

}

print(x)

}

groupscolored <- colorgroups(PC)

# plot using any plot function, but for the argument "col=" use "col=groupscolored

# PC1 vs PC2

plot (groupscolored[,15],groupscolored[,16], col=groupscolored[,18],

main = "GROUP: 8″, xlab="PC1", ylab="PC2",pch=16)

# PC1 vs PC3

plot (groupscolored[,15],groupscolored[,17], col=groupscolored[,18],

main = "GROUP: 8″, xlab="PC1", ylab="PC3",pch=16)

# PC2 vs PC3

plot (groupscolored[,16],groupscolored[,17], col=groupscolored[,18],

main = "GROUP: 8″, xlab="PC2", ylab="PC3",pch=16)

plot3d (groupscolored[,15],groupscolored[,16],groupscolored[,17], col=groupscolored[,18],

xlab="PC1", ylab="PC2", zlab="PC3", size=8)

grid3d ("x", at = NULL, col = "gray", lwd = 1, lty = 1, *n* = 5)

grid3d ("*y*+", at = NULL, col = "gray", lwd = 1, lty = 1, *n* = 5)

grid3d ("z", at = NULL, col = "gray", lwd = 1, lty = 1, *n* = 5)

title3d("GROUP of 8″, col = 'red', line = 3)

# rgl snapshot and pdf vector

rgl.snapshot("GROUP_8.png", fmt="png")

rgl.postscript("GROUP_8.pdf", "pdf", drawText = TRUE)

#——————————————————————–

# GROUP: 9

#——————————————————————–

#color coding groups of 9

colorgroups<-function(PC){

Group= vector(length=nrow(PC))

*x*<-cbind(PC,Group)

for(i in 1:nrow(x)){

# Number of if statements = Number of groups

if(x[*i*,8]==1) {x[*i*,18]<-"yellow"}

if(x[*i*,8]==2) {x[*i*,18]<-"blue"}

if(x[*i*,8]==3) {x[*i*,18]<-"red"}

if(x[*i*,8]==4) {x[*i*,18]<-"green"}

if(x[*i*,8]==5) {x[*i*,18]<-"orange"}

if(x[*i*,8]==6) {x[*i*,18]<-"purple"}

if(x[*i*,8]==7) {x[*i*,18]<-"deeppink"}

if(x[*i*,8]==8) {x[*i*,18]<-"chocolate"}

if(x[*i*,8]==9) {x[*i*,18]<-"gold"}

}

print(x)

}

groupscolored <- colorgroups(PC)

# plot using any plot function, but for the argument "col=" use "col=groupscolored

# PC1 vs PC2

plot (groupscolored[,15],groupscolored[,16], col=groupscolored[,18],

main = "GROUP: 9″, xlab="PC1", ylab="PC2",pch=16)

# PC1 vs PC3

plot (groupscolored[,15],groupscolored[,17], col=groupscolored[,18],

main = "GROUP: 9″, xlab="PC1", ylab="PC3",pch=16)

# PC2 vs PC3

plot (groupscolored[,16],groupscolored[,17], col=groupscolored[,18],

main = "GROUP: 9″, xlab="PC2", ylab="PC3",pch=16)

plot3d (groupscolored[,15],groupscolored[,16],groupscolored[,17], col=groupscolored[,18],

xlab="PC1", ylab="PC2", zlab="PC3", pch=16, size=8)

grid3d ("x", at = NULL, col = "gray", lwd = 1, lty = 1, *n* = 5)

grid3d ("*y*+", at = NULL, col = "gray", lwd = 1, lty = 1, *n* = 5)

grid3d ("z", at = NULL, col = "gray", lwd = 1, lty = 1, *n* = 5)

title3d("GROUP of 9″, col = 'red', line = 3)

# rgl snapshot and pdf vector

rgl.snapshot("GROUP_9.png", fmt="png")

rgl.postscript("GROUP_9.pdf", "pdf", drawText = TRUE)

#——————————————————————–

# GROUP: 10

#——————————————————————–

#color coding groups of 10

colorgroups<-function(PC){

Group= vector(length=nrow(PC))

*x*<-cbind(PC,Group)

for(i in 1:nrow(x)){

# Number of if statements = Number of groups

if(x[i,9]==1) {x[i,18]<-"yellow"}

if(x[i,9]==2) {x[i,18]<-"blue"}

if(x[i,9]==3) {x[i,18]<-"red"}

if(x[i,9]==4) {x[i,18]<-"green"}

if(x[i,9]==5) {x[i,18]<-"orange"}

if(x[i,9]==6) {x[i,18]<-"purple"}

if(x[i,9]==7) {x[i,18]<-"deeppink"}

if(x[i,9]==8) {x[i,18]<-"chocolate"}

if(x[i,9]==9) {x[i,18]<-"gold"}

if(x[i,9]==10) {x[i,18]<-"cyan"}

}

print(x)

}

groupscolored <- colorgroups(PC)

# plot using any plot function, but for the argument "col=" use "col=groupscolored

# PC1 vs PC2

plot (groupscolored[,15],groupscolored[,16], col=groupscolored[,18],

main = "GROUP: 10″, xlab="PC1", ylab="PC2",pch=16)

# PC1 vs PC3

plot (groupscolored[,15],groupscolored[,17], col=groupscolored[,18],

main = "GROUP: 10″, xlab="PC1", ylab="PC3",pch=16)

# PC2 vs PC3

plot (groupscolored[,16],groupscolored[,17], col=groupscolored[,18],

main = "GROUP: 10″, xlab="PC2", ylab="PC3",pch=16)

plot3d (groupscolored[,15],groupscolored[,16],groupscolored[,17], col=groupscolored[,18],

xlab="PC1", ylab="PC2", zlab="PC3", pch=16, size=8)

grid3d ("x", at = NULL, col = "gray", lwd = 1, lty = 1, *n* = 5)

grid3d ("*y*+", at = NULL, col = "gray", lwd = 1, lty = 1, *n* = 5)

grid3d ("z", at = NULL, col = "gray", lwd = 1, lty = 1, *n* = 5)

title3d("GROUP of 10″, col = 'red', line = 3)

# rgl snapshot and pdf vector

rgl.snapshot("GROUP_10.png", fmt="png")

rgl.postscript("GROUP_10.pdf", "pdf", drawText = TRUE)

#——————————————————————–

# GROUP: 11

#——————————————————————–

#color coding groups of 11

colorgroups<-function(PC){

Group= vector(length=nrow(PC))

*x*<-cbind(PC,Group)

for(i in 1:nrow(x)){

# Number of if statements = Number of groups

if(x[i,10]==1) {x[i,18]<-"yellow"}

if(x[i,10]==2) {x[i,18]<-"blue"}

if(x[i,10]==3) {x[i,18]<-"red"}

if(x[i,10]==4) {x[i,18]<-"green"}

if(x[i,10]==5) {x[i,18]<-"orange"}

if(x[i,10]==6) {x[i,18]<-"purple"}

if(x[i,10]==7) {x[i,18]<-"deeppink"}

if(x[i,10]==8) {x[i,18]<-"chocolate"}

if(x[i,10]==9) {x[i,18]<-"gold"}

if(x[i,10]==10) {x[i,18]<-"cyan"}

if(x[i,10]==11) {x[i,18]<-"darkkhaki"}

}

print(x)

}

groupscolored <- colorgroups(PC)

# plot using any plot function, but for the argument "col=" use "col=groupscolored

# PC1 vs PC2

plot (groupscolored[,15],groupscolored[,16], col=groupscolored[,18],

main = "GROUP: 11″, xlab="PC1", ylab="PC2",pch=16)

# PC1 vs PC3

plot (groupscolored[,15],groupscolored[,17], col=groupscolored[,18],

main = "GROUP: 11″, xlab="PC1", ylab="PC3",pch=16)

# PC2 vs PC3

plot (groupscolored[,16],groupscolored[,17], col=groupscolored[,18],

main = "GROUP: 11″, xlab="PC2", ylab="PC3",pch=16)

plot3d (groupscolored[,15],groupscolored[,16],groupscolored[,17], col=groupscolored[,18],

xlab="PC1", ylab="PC2", zlab="PC3", pch=16, size=8)

grid3d ("x", at = NULL, col = "gray", lwd = 1, lty = 1, *n* = 5)

grid3d ("*y*+", at = NULL, col = "gray", lwd = 1, lty = 1, *n* = 5)

grid3d ("z", at = NULL, col = "gray", lwd = 1, lty = 1, *n* = 5)

title3d("GROUP of 11″, col = 'red', line = 3)

# rgl snapshot and pdf vector

rgl.snapshot("GROUP_11.png", fmt="png")

rgl.postscript("GROUP_11.pdf", "pdf", drawText = TRUE)

#——————————————————————–

# GROUP: 12

#——————————————————————–

#color coding groups of 12

colorgroups<-function(PC){

Group= vector(length=nrow(PC))

*x*<-cbind(PC,Group)

for(i in 1:nrow(x)){

# Number of if statements = Number of groups

if(x[i,11]==1) {x[i,18]<-"yellow"}

if(x[i,11]==2) {x[i,18]<-"blue"}

if(x[i,11]==3) {x[i,18]<-"red"}

if(x[i,11]==4) {x[i,18]<-"green"}

if(x[i,11]==5) {x[i,18]<-"orange"}

if(x[i,11]==6) {x[i,18]<-"purple"}

if(x[i,11]==7) {x[i,18]<-"deeppink"}

if(x[i,11]==8) {x[i,18]<-"chocolate"}

if(x[i,11]==9) {x[i,18]<-"gold"}

if(x[i,11]==10) {x[i,18]<-"cyan"}

if(x[i,11]==11) {x[i,18]<-"darkkhaki"}

if(x[i,11]==12) {x[i,18]<-"sienna"}

}

print(x)

}

groupscolored <- colorgroups(PC)

# plot using any plot function, but for the argument "col=" use "col=groupscolored

# PC1 vs PC2

plot (groupscolored[,15],groupscolored[,16], col=groupscolored[,18],

main = "GROUP: 12″, xlab="PC1", ylab="PC2",pch=16)

# PC1 vs PC3

plot (groupscolored[,15],groupscolored[,17], col=groupscolored[,18],

main = "GROUP: 12″, xlab="PC1", ylab="PC3",pch=16)

# PC2 vs PC3

plot (groupscolored[,16],groupscolored[,17], col=groupscolored[,18],

main = "GROUP: 12″, xlab="PC2", ylab="PC3",pch=16)

plot3d (groupscolored[,15],groupscolored[,16],groupscolored[,17], col=groupscolored[,18],

xlab="PC1", ylab="PC2", zlab="PC3", pch=16, size=8)

grid3d ("x", at = NULL, col = "gray", lwd = 1, lty = 1, *n* = 5)

grid3d ("*y*+", at = NULL, col = "gray", lwd = 1, lty = 1, *n* = 5)

grid3d ("z", at = NULL, col = "gray", lwd = 1, lty = 1, *n* = 5)

title3d("GROUP of 12″, col = 'red', line = 3)

# rgl snapshot and pdf vector

rgl.snapshot("GROUP_12.png", fmt="png")

rgl.postscript("GROUP_12.pdf", "pdf", drawText = TRUE)

#——————————————————————–

# GROUP: 13

#——————————————————————–

#color coding groups of 13

colorgroups<-function(PC){

Group= vector(length=nrow(PC))

*x*<-cbind(PC,Group)

for(i in 1:nrow(x)){

# Number of if statements = Number of groups

if(x[i,12]==1) {x[i,18]<-"yellow"}

if(x[i,12]==2) {x[i,18]<-"blue"}

if(x[i,12]==3) {x[i,18]<-"red"}

if(x[i,12]==4) {x[i,18]<-"green"}

if(x[i,12]==5) {x[i,18]<-"orange"}

if(x[i,12]==6) {x[i,18]<-"purple"}

if(x[i,12]==7) {x[i,18]<-"deeppink"}

if(x[i,12]==8) {x[i,18]<-"chocolate"}

if(x[i,12]==9) {x[i,18]<-"gold"}

if(x[i,12]==10) {x[i,18]<-"cyan"}

if(x[i,12]==11) {x[i,18]<-"darkkhaki"}

if(x[i,12]==12) {x[i,18]<-"sienna"}

if(x[i,12]==13) {x[i,18]<-"indianred"}

}

print(x)

}

groupscolored <- colorgroups(PC)

# plot using any plot function, but for the argument "col=" use "col=groupscolored

# PC1 vs PC2

plot (groupscolored[,15],groupscolored[,16], col=groupscolored[,18],

main = "GROUP: 13″, xlab="PC1", ylab="PC2",pch=16)

# PC1 vs PC3

plot (groupscolored[,15],groupscolored[,17], col=groupscolored[,18],

main = "GROUP: 13″, xlab="PC1", ylab="PC3",pch=16)

# PC2 vs PC3

plot (groupscolored[,16],groupscolored[,17], col=groupscolored[,18],

main = "GROUP: 13″, xlab="PC2", ylab="PC3",pch=16)

plot3d (groupscolored[,15],groupscolored[,16],groupscolored[,17], col=groupscolored[,18],

xlab="PC1", ylab="PC2", zlab="PC3", pch=16, size=8)

grid3d ("x", at = NULL, col = "gray", lwd = 1, lty = 1, *n* = 5)

grid3d ("*y*+", at = NULL, col = "gray", lwd = 1, lty = 1, *n* = 5)

grid3d ("z", at = NULL, col = "gray", lwd = 1, lty = 1, *n* = 5)

title3d("GROUP of 13″, col = 'red', line = 3)

# rgl snapshot and pdf vector

rgl.snapshot("GROUP_13.png", fmt="png")

rgl.postscript("GROUP_13.pdf", "pdf", drawText = TRUE)

#——————————————————————–

# GROUP: 14

#——————————————————————–

#color coding groups of 14

colorgroups<-function(PC){

Group= vector(length=nrow(PC))

*x*<-cbind(PC,Group)

for(i in 1:nrow(x)){

# Number of if statements = Number of groups

if(x[i,13]==1) {x[i,18]<-"yellow"}

if(x[i,13]==2) {x[i,18]<-"blue"}

if(x[i,13]==3) {x[i,18]<-"red"}

if(x[i,13]==4) {x[i,18]<-"green"}

if(x[i,13]==5) {x[i,18]<-"orange"}

if(x[i,13]==6) {x[i,18]<-"purple"}

if(x[i,13]==7) {x[i,18]<-"deeppink"}

if(x[i,13]==8) {x[i,18]<-"chocolate"}

if(x[i,13]==9) {x[i,18]<-"gold"}

if(x[i,13]==10) {x[i,18]<-"cyan"}

if(x[i,13]==11) {x[i,18]<-"darkkhaki"}

if(x[i,13]==12) {x[i,18]<-"sienna"}

if(x[i,13]==13) {x[i,18]<-"indianred"}

if(x[i,13]==14) {x[i,18]<-"khaki"}

}

print(x)

}

groupscolored <- colorgroups(PC)

# plot using any plot function, but for the argument "col=" use "col=groupscolored

# PC1 vs PC2

plot (groupscolored[,15],groupscolored[,16], col=groupscolored[,18],

main = "", xlab="PC1", ylab="PC2",pch=16)

# PC1 vs PC3

plot (groupscolored[,15],groupscolored[,17], col=groupscolored[,18],

main = "GROUP: 14″, xlab="PC1", ylab="PC3",pch=16)

# PC2 vs PC3

plot (groupscolored[,16],groupscolored[,17], col=groupscolored[,18],

main = "GROUP: 14″, xlab="PC2", ylab="PC3",pch=16)

plot3d (groupscolored[,15],groupscolored[,16],groupscolored[,17], col=groupscolored[,18],

xlab="PC1", ylab="PC2", zlab="PC3", pch=16, size=8)

grid3d ("x", at = NULL, col = "gray", lwd = 1, lty = 1, *n* = 5)

grid3d ("*y*+", at = NULL, col = "gray", lwd = 1, lty = 1, *n* = 5)

grid3d ("z", at = NULL, col = "gray", lwd = 1, lty = 1, *n* = 5)

title3d("GROUP of 14″, col = 'red', line = 3)

# rgl snapshot and pdf vector

rgl.snapshot("GROUP_14.png", fmt="png")

rgl.postscript("GROUP_14.pdf", "pdf", drawText = TRUE)

#——————————————————————–

# GROUP: 15

#——————————————————————–

#color coding groups of 15

colorgroups<-function(PC){

Group= vector(length=nrow(PC))

*x*<-cbind(PC,Group)

for(i in 1:nrow(x)){

# Number of if statements = Number of groups

if(x[i,14]==1) {x[i,18]<-"yellow"}

if(x[i,14]==2) {x[i,18]<-"blue"}

if(x[i,14]==3) {x[i,18]<-"red"}

if(x[i,14]==4) {x[i,18]<-"green"}

if(x[i,14]==5) {x[i,18]<-"orange"}

if(x[i,14]==6) {x[i,18]<-"purple"}

if(x[i,14]==7) {x[i,18]<-"deeppink"}

if(x[i,14]==8) {x[i,18]<-"chocolate"}

if(x[i,14]==9) {x[i,18]<-"gold"}

if(x[i,14]==10) {x[i,18]<-"cyan"}

if(x[i,14]==11) {x[i,18]<-"darkkhaki"}

if(x[i,14]==12) {x[i,18]<-"sienna"}

if(x[i,14]==13) {x[i,18]<-"indianred"}

if(x[i,14]==14) {x[i,18]<-"khaki"}

if(x[i,14]==15) {x[i,18]<-"lightblue"}

}

print(x)

}

groupscolored <- colorgroups(PC)

# plot using any plot function, but for the argument "col=" use "col=groupscolored

# PC1 vs PC2

plot (groupscolored[,15],groupscolored[,16], col=groupscolored[,18],

main = "GROUP: 15″, xlab="PC1", ylab="PC2",pch=16)

# PC1 vs PC3

plot (groupscolored[,15],groupscolored[,17], col=groupscolored[,18],

main = "GROUP: 15″, xlab="PC1", ylab="PC3",pch=16)

# PC2 vs PC3

plot (groupscolored[,16],groupscolored[,17], col=groupscolored[,18],

main = "GROUP: 15″, xlab="PC2", ylab="PC3",pch=16)

plot3d (groupscolored[,15],groupscolored[,16],groupscolored[,17], col=groupscolored[,18],

xlab="PC1", ylab="PC2", zlab="PC3", pch=16, size=8)

grid3d ("x", at = NULL, col = "gray", lwd = 1, lty = 1, *n* = 5)

grid3d ("*y*+", at = NULL, col = "gray", lwd = 1, lty = 1, *n* = 5)

grid3d ("z", at = NULL, col = "gray", lwd = 1, lty = 1, *n* = 5)

title3d("GROUP of 15″, col = 'red', line = 3)

# rgl snapshot and pdf vector

rgl.snapshot("GROUP_15.png", fmt="png")

rgl.postscript("GROUP_15.pdf", "pdf", drawText = TRUE)

#———————————————————————

## Age statistics

### Local glacial stages

True moraine ages or local glacial stages (e.g., Saha et al. [3]) may be assessed using a variety of statistical methods. For example, reduced chi-squared (χ^2^) statistics (or mean square weighted deviation [MSWD]) is often applied to assess the distribution of apparent moraine ages [17,18]. Any age population with χ^2^ > 1 indicates poor goodness of fit indicating large scatter/outliers; χ^2^ > 1 but <95% confidence interval indicates acceptable outliers or acceptable goodness of fit between observations and estimates. Outliers may also be detected and removed from the age population using other statistical techniques, including Chauvenet's criterion [19]. Since numerical ages are assumed to have normal distribution probabilities, Chauvenet's criterion is a powerful tool. Other quantitative methods commonly applied to detect outliers, but not shown here, include skewness [17,18], probability density estimates [20], two standard deviations from the mean [21], two mean absolute deviations from the median [22], and generalized extreme Students deviates [23]. We encourage the readers to explore these methods as well to cross-validate their results. However, note that these are entirely statistical treatments that do not consider the background geological conditions. We therefore argue that readers must evaluate their statistically detected outliers with detail field observations before removing them from further analysis [3].

**Fig. 4:**
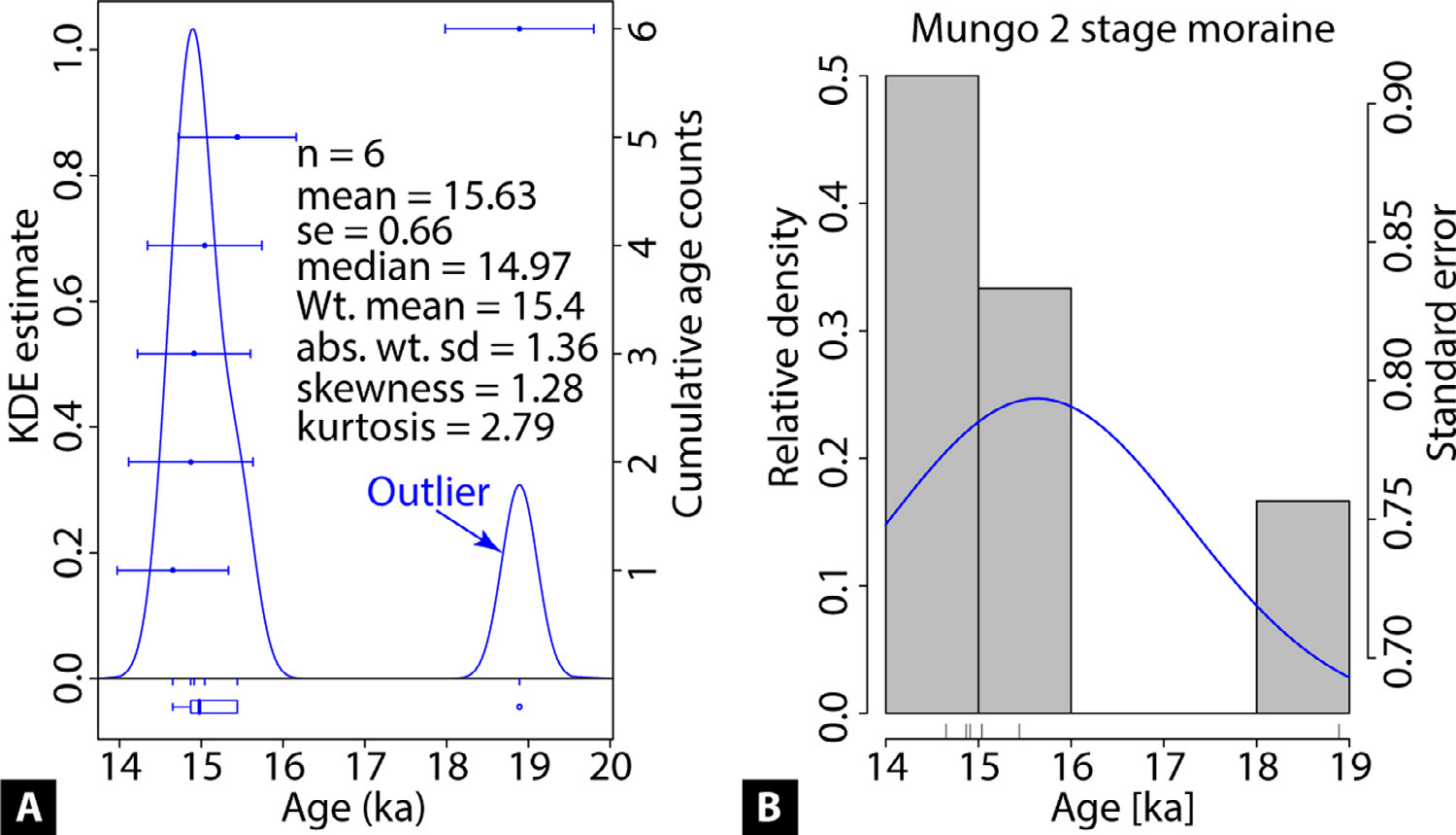
Kernel density estimation [KDE] (A) and histogram (B) showing the distribution of ^10^Be ages. The six ages [14.7 ± 0.7, 14.9 ± 0.7, 15.0 ± 0.7, 14.9 ± 0.8, 18.9 ± 0.9, 15.4 ± 0.7] are compiled from the Mungo 2 stage moraine after Seong et al. [24]. The sample age with χ^2^ > 2 (i.e., ~19 ka) is identified as an outlier and must be removed from further analysis. Here, the summary statistics include the outlier. Readers must note that the summary statistics must be used on outlier free distribution for publication purposes.

Outlier free apparent moraine ages may be used to estimate mean moraine ages or true moraine ages [17,18] or local glacial stages [3]. Several popular measures of central values are the arithmetic mean ± standard deviation (sd), weighted mean ± weighted sd, and median ± sd (Fig. 4) [17,18]. The apparent ages can be visualized through the kernel density (relative probability) plots and histogram (Fig. 4). Additionally, skewness and kurtosis may also be useful to infer whether ages have longer tail distribution or positively skewed (possibly inheritance) or younger tail distribution or negatively skewed (possibly incomplete exposure) (see [17], [18] for further explanation).

Note that most of the statistical methods stated here work for ≥3 apparent ages on a moraine. Since chronological studies often use limited discrete ages/samples, cautioned must be performed in applying these statistical techniques and interpreting the ages. Below is a compilation of the statistical methods stated above in R modified from different sources. For example, the χ^2^ test is modified after Applegate et al. [18]; Chauvenet's criterion after Taylor [19]; R luminescence package from https://CRAN.R-project.org/ package=Luminescence. Supplementary Table S2 can be used as a template.

#————————————————————–

# Reduced Chi-Square test

# Setting up the directory and file input

## "Directory need to change if using in another computer"

setwd("C:/users/folder_name/")

# File input; use template S2

data<-read.csv(file.choose()) # Read the CSV file

head(data) # For a quick look of the file to see the variables

#——————————————————-

# Reduced Chi-Square test (X^2-squared test) modified after Applegate et al. (2012)

age.mean= mean(data$Age)

chi.squared= (1/(length(data$Age)-1))*(sum((data$Age-age.mean)^2/(data$Err)^2))

print(chi.squared) # Read from the console

# If the X-squared value is >1, check for outliers

#——————————————————-

# Outliers detection using Chauvenet's criterion after Taylor (1997)

# A minimum of 3 data (n) requires performing this technique

Chauvenet=sqrt((((data$Age-(mean(data$Age)))/sd(data$Age)))^2)

print(Chauvenet) # Read from the console if limited n is used

print(length(data$Age)) # Look at the Chauvenet's table

# Otherwise save as csv file

write.table(Chauvenet, " C:/users/folder_name/Chauvenet.csv", sep=",")

# Look at the Chauvenet's table at https://www.statisticshowto.datasciencecentral.com/chauvenets-criterion/

# Any value higher than the tabulated value may be regarded as outliers

# Remove the outliers, repeat the chi-squared test and Chauvenet's criterion

#—————————————————————

# Check for age distribution in kernel density estimation [KDE] & histogram with summary statistics (Fig. 4A)

# Install necessary packages @ https://CRAN.R-project.org/package=Luminescence

install.packages("Luminescence")

library(Luminescence)

# Plot the kernel density estimate with summary statistics

KDE_out <-plot_KDE(data = data, main = " Age distribution [ka]",

xlab = " Age [ka]",

ylab = *c*("KDE estimate", "Cumulative age counts"),

boxplot = TRUE,

summary = *c*("n","mean","se.abs","median","skewness","kurtosis"),

summary.pos = "topleft",

col = *c*("blue", "orange"),

output = TRUE)

#——————————————————-

# Plot histogram with summary statistics (Fig. 4B)

plot_Histogram(data,

rug = TRUE,

normal_curve = TRUE,

cex.global = 1,

pch = 1,

color = *c*("gray", "black", "blue", "green"),

summary = *c*("n","mean.weighted","sdabs.weighted"),

summary.pos = "topleft",

main = "Histogram of apparent ages",

mtext = "XX stage moraine",

xlab= *c*("Age [ka]"),

ylab = *c*("Relative density",

"Standard error"))

#——————————————————-

### Regional glacial stages

**Fig. 5:**
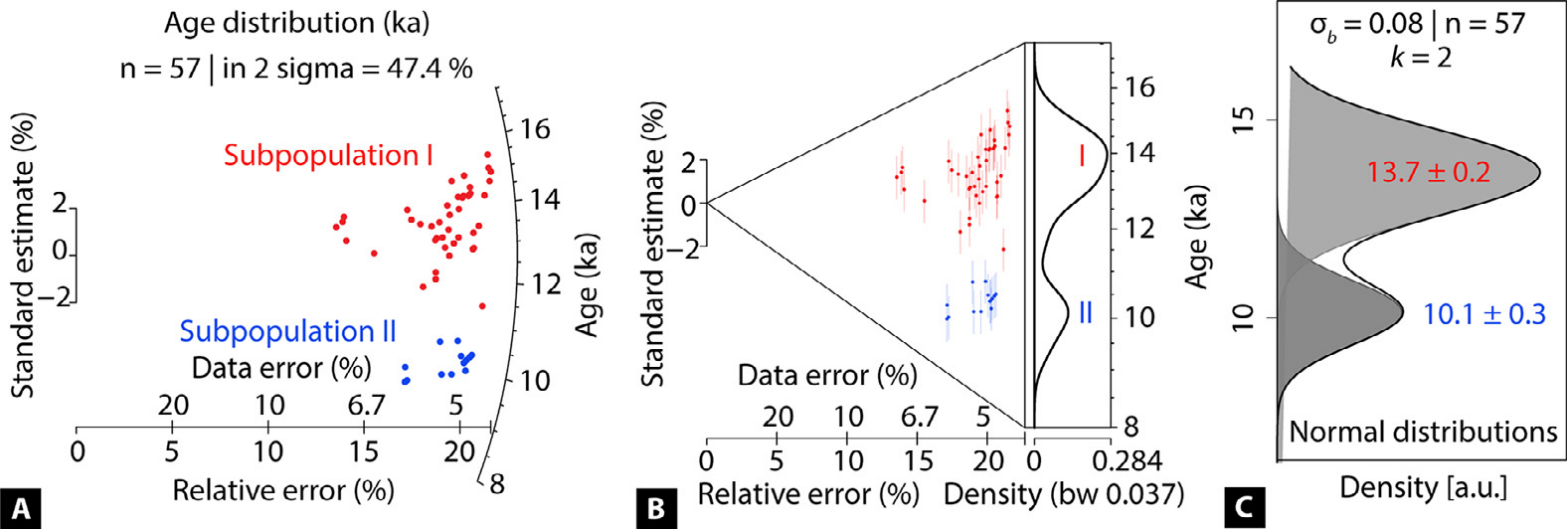
Plots displaying local stage/age distribution and discrete subpopulations. Subpopulations are color-coded separately to highlight them in the plots. (A) Radial plotter, (B) Abanico plot, and (C) Finite mixture model (FMM) all showing the two distinct age subpopulations or local stages. Age data are derived from Table 5 of Saha et al. [3] (i.e., the first two local glacial stages in climatic zone 1a).

Once local glacial stages are defined and organized according to the climatic groups/zones discussed in Section 1, discrete regional glacial stages may be identified using a variety of visual and statistical techniques. For example, Radial plots (Fig. 5A) [25] and Abanico plots (Fig 5B) [26] are visually useful to identify distinct subpopulations. These subpopulations can also be detected quantitatively using the finite mixture model (Fig. 5C) [25,27] and Student's *t*-test [28]. The following R codes incorporate these visual and statistical tools [3]. Use Table S2 as a template where the first column is the mean moraine age in ka, and the 2nd column is ± 1σ (internal error) in ka.

Additional useful methods may include the probability density function of Dortch et al. [2] (not shown here). We evaluated our results with the PDF techniques of Dortch et al. [2] (see Fig. S9 in Saha et al. [3]) and found no significant difference.

# Setting up the directory and file input

## "Directory need to change if using in other computer"

setwd("C:/users/folder_name/")

# file input

# AGES ARE IN KA (i.e., 1000 years)

data<-read.csv(file.choose()) # read the csv file;

head(data) # for quick look of the file to see the variables

#———————————————————————

#install package "Luminescence"

install.packages("Luminescence")

library(Luminescence) #load luminescence package

#———————————————————————

# Visual method

# Radial plotter (Galbraith et al., 1999) (Fig. 5A)

plot_RadialPlot(data = data, grid.col = "none", y.ticks = TRUE,

output = TRUE, lwd = 0,

main = " Age distribution (ka)",

xlab = *c*("Data error (%)", "Relative error (%)"),

ylab = "Standard estimate (%)",

zlab = "Age (ka)", summary = *c*("n", "in.2s"))

#———————————————————————

# Abanico Plot (Burow, 2018) using the same data (Fig. 5B)

plot_AbanicoPlot(data = data, bar.col = FALSE, grid.col = "none",

main = " Age distribution (ka)",

xlab = *c*("Data error (%)", "Relative error (%)"),

ylab = "Standard estimate (%)", zlab = "Age (ka)",

y.axis = TRUE,

error.bars = TRUE)

#———————————————————————

# Statistical models

# Finite mixture model (FMM; Galbraith et al., 1999) (Fig. 5C)

# FMM works better when ages are in years

samp.data=(data *1000) #changing the age data from ka to a (i.e., years)

head(samp.data)

# Note: Change the "dose.scale" to have your appropriate age range; here 0 to 18,000 years

# Change "sigmab"; low value for finer resolution and high for coarse resolution

# A warning massage mean your "sigmab" value is either two low or too high (try changing it)

# Here we used a conservative 8% (can be changed based on the data)

# Try using a consistent "sigmab" value between regional stages for consistency

# n.components can also be varied based on number of possible supopultaion and BIC score

calc_FiniteMixture(data=samp.data, sigmab=0.08, n.components=*c*(2:4), grain.probability = TRUE,

dose.scale=*c*(0, 18,000), pdf.weight = TRUE, main = " Age distribution (yr)")

# Lowest BIC score for k should be used to differentiate regional glacial stages

#—————————————————————

#————————————————————–

# Fisher's F-test (may be tested before *t*-test; optional)

# F-test indicates the homogeneity between two variances

# Setting up the directory and file input

## "Directory need to change if using in another computer"

setwd("C:/users/folder_name/")

# File input; Supplementary table S2 is used as a template

samp<-read.csv(file.choose()) # Read the CSV file

head(samp) # For a quick look of the file to see the variables

# Local stages/mean moraine ages are Separated based on above methods

# Manually assign the range

# Group 1 —————————————————-

group1= (samp[1:50, 1]) # Rows/groups must be assigned based on above methods

NewOld1= ((samp[1:50, 1])+(samp[1:50, 2])) # Rows/groups max range; enter the range manually

NewYoung1= ((samp[1:50, 1])-(samp[1:50, 2])) # Rows/groups min range; enter the range manually

# Binding the max and min ages together to the mean moraine ages

group1<- rbind(group1, NewOld1)

group1<- rbind(group1, NewYoung1)

# Group 2 —————————————————-

group2= (samp[51:64, 1]) # Rows/groups range must be assigned based on above methods

NewOld2= ((samp[51:64, 1])+(samp[51:64, 1])) # Rows/groups max range; enter the range manually

NewYoung2= ((samp[51:64, 1])-(samp[51:64, 1])) # Rows/groups min range; enter the range manually

# Binding the max and min ages together to the mean moraine ages

group2<- rbind(group2, NewOld2)

group2<- rbind(group2, NewYoung2)

#————————————————————-

# Fisher's F-test to compare two groups

var.test(group2, group1) # record the result from the console

# If p-value is <0.05, we can tentatively assume that two groups are not homogeneous

# However, a best way to check our results is by looking at the tabulated F-value

# To get the tabulated F-value, using 95% confidence interval,

# degrees of freedom of numerator=13, and degrees of freedom of denominator=49 (read from console)

qf(0.95, 41, 199)

# If the computed F-value (i.e., var.test) > tabulated F-value, groups are not homogeneous

#————————————————————-

# Two-sample Student's *t*-test (Edgell and Noon, 1984)

t.test(group1,group2, var.equal=TRUE, paired=FALSE)

# If p-value <0.05, averages of the two groups are significantly dissimilar

# The best way to check our results is by looking at the tabulated t-value

# To get the tabulated t-value, using 97.5% confidence interval, degree of freedom = 62 (read from console)

qt(0.975, 240)

# If computed t-value > tabulated t-value, groups are dissimilar

#————————————————————–

## Morphometric models

### Equilibrium-line altitudes (ELAs) and glacier hypsometry

Osmaston [29] detailed the steps to estimate ELAs using area-altitude (AA), area accumulation ratio (AAR), and toe-headwall accumulation ratio (THAR) methods. Choosing the correct ratios (or a combination) for AAR and THAR are dependent primarily, among other things, on the topography, size of the glacier, and microclimate [30]. Saha et al. [3], e.g., obtained AAR and THAR ratios from literature for each climatic group (see Section 3.3 in Saha et al. [3] for details).

**Fig. 6:**
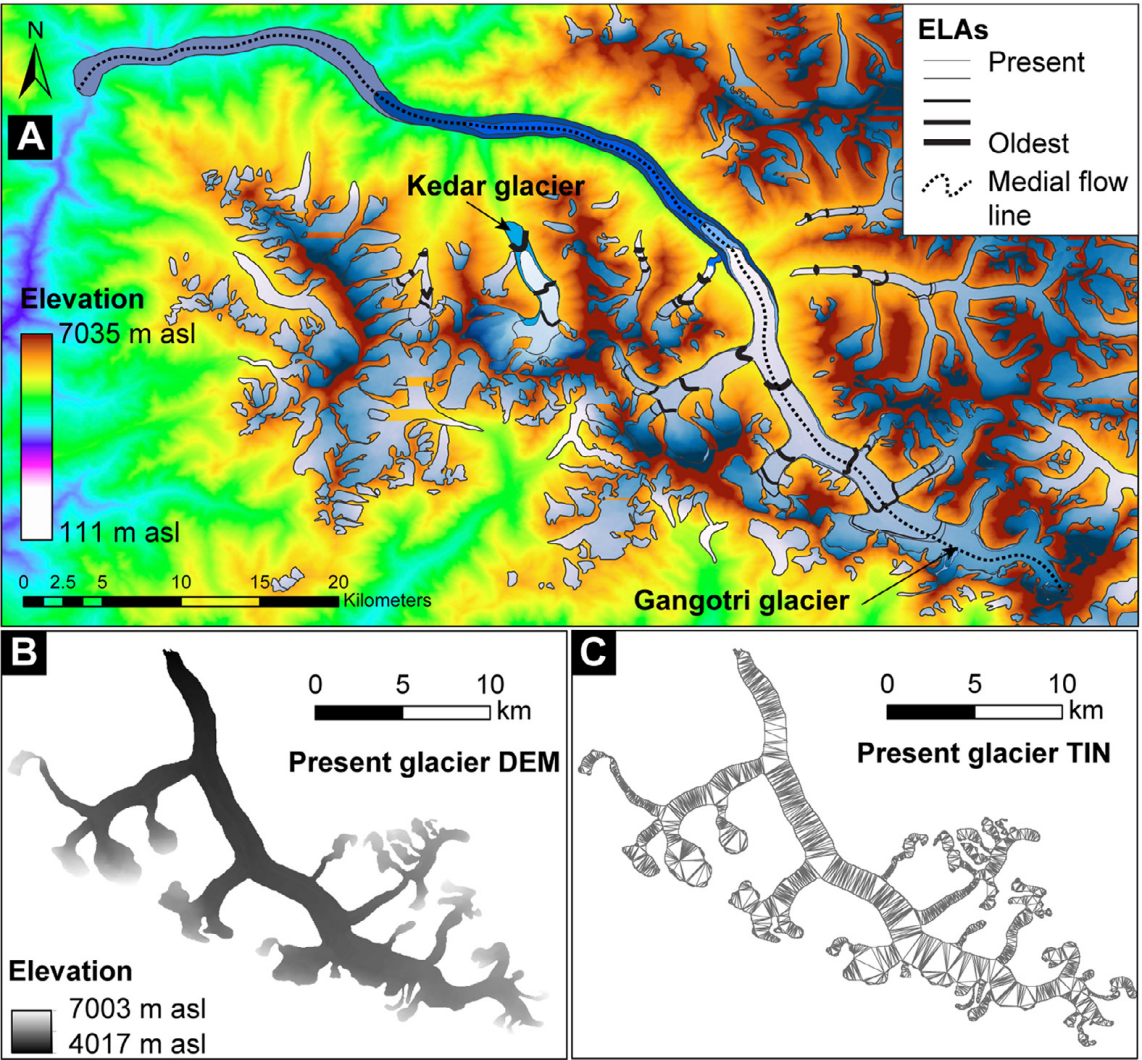
An example of mapped glaciated areas (superimposed on the digital elevation model) of Gangotri glacier in the Bhagirathi valley, India, are shown for estimating equilibrium-line altitudes (ELAs). (A) Glaciated areas are mapped from Google Earth and Landsat ETM+ images and displayed from present to the oldest preserved glacier advance. ELAs for each glacier are shown in solid black lines; the thickness of the ELA lines varies from youngest to the oldest. The medial glacier flow length is measured along the dotted black line for each local glacier stage/advance. (B) An example of the extracted DEM of the present glacier using the mapped area (polygon). (C) An example of the converted triangular irregular networks (TIN) of the present glacier from the extracted DEM.

We mapped present and past glaciated areas from Google Earth and Landsat ETM+ images in ArcGIS 10.5 (Fig. 6A; see also supplementary file S3) [31]. Those maps (vector layers) were used to extract Advanced Spaceborne Thermal Emission and Reflection Radiometer (ASTER) global DEMs using the ‘Extract by Mask’ tool in ArcGIS 10.5 (Fig. 6B). Extracted (raster) DEMs were converted into ASCII files to aid the subsequent steps. The raster DEMs were also converted into triangular irregular networks (‘TIN’ in ArcGIS) layers to calculate the 3D surfaces of glaciers (Fig. 6C). Glaciated surface areas were estimated using the TIN layer and ‘Interpolate Polygon to Multipatch’ tool in ArcGIS. We also generated glacier's hypsometry [32] using the previously generated ASCII file (see supplementary Fig. S5 in Saha et al. [3]). The Read ArcGrid program developed by Professor David Nash of the University of Cincinnati was used to generate the glacier's hypsometry. The Read ArcGrid program is a simple java program that calculates the Elevation Relief Ratio (hypsometric integral) for a matrix of elevations using Pike and Wilson's method [32]. The hypsometric integral data and glaciated surface area can be directly copied and pasted into the ELAs excel file (highlighted in yellow in the supplementary Table S4). The excel file (Table S4 ELA calculation table) is prepared using the steps and recommendations of Osmaston [29].

### The linear inverse glacier flow model

We reconstructed paleotemperature changes relative to present from the linear change in glacier length using the first-order glacier dynamics model of Oerlemans [33,34] for each glacier. Since this simple linear inverse glacier flow model requires a limited number of variables, it is easy to reconstruct for a wide range of Central Asian glaciers. For example, the essential model variables are: present glacier (medial) length (L_0_ in m), past (medial) lengths (L in m) based on the maximum extent of preserved moraines in the valley (Fig. 6A), mean slope of the glacier surface (s in%), present mean annual precipitation (P in m/year), time of moraine formation (t in a or year), and age uncertainties (e in a or year). Since the linear flow model considers climate sensitivity (*с*) and response time (τ) of the glacier to estimate the lag time, we argue that the model is a decent approximation for widely different climatic regions throughout the orogen.

Climate sensitivity (*c*) is calculated as *c* = 2.3*P*^0.6^*s*^−1^ (x) and response time (τ) as τ = 13.6β^−1^*s*^−1^(1 + 20 s)^−1/2^*L'*^−1/2^ (xi)

Here, *P* is the annual climatological precipitation in m *a*^−1^, s is the mean surface slope of the glacier in percent, *L'* is the present glacier length in m calculated along the medial flow line of the glacier (Fig. 6A), and β is the altitudinal mass-balance gradient, which is defined as β = 0.006*P*^1/2^ [33,34]. Note that these formulas and constants are obtained by calibrating a simple model of glacier dynamics against globally distributed glacier records [33]. We are unable to calibrate the constants for our study areas due to the lack of detailed (proxy or instrumental) long-term glacier length change and climatological data in the higher reaches. However, we argue that the (globally calibrated) inverse linear flow model is a simple first-order representation of the glacier length change with a corresponding change in temperature (*T*), given the climate sensitivity and the response time (i.e., the lag time) of the glacier. Since we have averaged our reconstructed *T* regionally and have also provided ±30% change in *P*, given the large uncertainty in *P* during the Holocene over the orogen, we argue that the changing pattern of reconstructed *T* is useful for our understanding, if not exact. We have derived *s* and *P* for each glacier from the ASTER GDEM and the TRMM gridded cells, respectively. The model assumes a steady-state scenario and a larger influence of ambient temperature (in K) in modulating the equilibrium glacier length on a continental scale [33,34]. The following is a MATLAB code of the linear inverse glacier flow model modified after Oerlemans [33,34].

clc

close all

%%—————-

% A linear inverse glacier flow-line model

% Reference: Oerlemans, 2001, pp-83-92 and Oerlemans, 2005

%%—————-

% Basic parameters of the glacier (needs to change for every new glacier)

% Present (reference) length of the glacier (m); manually input these data

L0= 1751;% need to change every time for new glacier

*s*= 0.45;% mean slope of the glacier (calculated using ArcGIS) in percent

*P*= 1.988;% climatological mean annual precipitation in m/year

% Basic data (dL & t) must be arranged from older to younger

dL=[9803, 8004, 7570, 3254, 3002, 1005, 505, 0];%length change from L0 in m

*t*=[10,032, 7900, 6055, 3423, 2450, 1036, 259, 0];% time parameter in years

*e*=[304, 204, 200, 128, 78, 90, 22, 0];%  Error in ages (year)

%%—————-

% Constant parameters

*n*=0.006;% relates the mean glacier thickness to the glacier length

*u*=2.3;% constant used in climate sensitivity

*a*=0.6;% constant used in climate sensitivity

*b*=13.6;% constant used in response time

*y*=20;% constant used in response time

beta= n*(P.^0.5); % annual mass balance gradient (m/ year)

%%—————-

% Llinear interpolation of dL

dt=10;

t_fine=1:dt:max(t);

dL_fine=interp1(t,dL,t_fine);%linear interpolation– works better

%%—————-

% Since the data is not smooth, we need to smooth the data using % Butterworth

[*bb,aa*]=butter(1,1500/10,000,'low');%low pass (must change and see how%%itbehaves with higher and lower values

dL_smooth=filter(bb,aa,dL_fine);

%%—————-

% To calculate dLdt: First step

% calculating dL difference per dt

dL_prime=diff(dL_smooth);%compute the difference in length

% MY WACKY FIX of the boundary condition (adding 0 in the 1st column)

dL_prime_ref=dL_prime;

dL_max=length(dL_smooth);

for *c*=2:dL_max

dL_prime(1,c)=dL_prime_ref(1,c-1);

end

dL_prime(1,1)=0;

%%—————-

% To calculate dLdt: Second step

% Now calcuting the required dL/dt, that is in m/year

dLdt=dL_prime/dt;

  

%%—————-

% Calculating change in temperature (in K) from glacier length

% Let's loop it

% dt=10;

for tm= 1:dt:max(t) % time loop

  

% calculating climate sensitivity (km/k)

*c*= u*P.^a/s;

  

% calculating glacier response time (year)

res_*t*= *b*./beta./s*(1+y*s)^-0.5*L0^-0.5; % calculating response time

  

% calculating change in temperature as a function of glacier geometry

*T*=-(1/c)*(dL_smooth+(res_t.*dLdt));

*T*=*T*/100;% had to introduce this value.

  

% plotting the data

figure(1)

% plotting the change in glacier length over time (m)

subplot(211);

yyaxis left

plot(t_fine,dL_fine,'-','MarkerEdgeColor','g');

hold on

plot(t_fine,dL_smooth, '–','MarkerEdgeColor','r');

ylabel('Relative difference in length (m)')

hold on

yyaxis right

plot(t_fine, dLdt,'–','MarkerEdgeColor','k')

grid on

ylabel('dLdt (m/a)')

xlabel('Time (years)')

title('Linear Inverse Glacier Flow Model')

hold off

  

% plotting the change in temperature over time (k)

subplot(2,1,2)

plot(t_fine, T,'–','MarkerEdgeColor', 'r', 'LineWidth', 2),

grid on

xlabel('Time (years)')

ylabel('Temperature (k)')

title('Reconstructed Temperature graph')

  

end    % end of the time loop

  

%%—————-

% Calculating mean and standard deviations for each Local glacial advance

% Creating a matrix of interpolated time and calculted temp. change

data=[*t*_*fine*;T];

dtm=10;% generally 10 because our dt=10

% now for the first age group

t1=(t(1,1)-e(1,1));

t2=max(t);

data1=data(:,((t1/dtm)+1):((t2/dtm)));

Mean1= mean(data1(2,:));

STD1=std(data1(2,:));

  

yneg=STD1;

ypos=STD1;

xneg=*e*(1,1);

xpos=0;

  

hold on

errorbar(max(t),Mean1,yneg,ypos,xneg,xpos)

plot(data1(1,:),data1(2,:), 'r','LineWidth', 2)

  

  

%for second age group

t3=(t(1,2)-e(1,2));

t4=(t(1,2)+*e*(1,2));

data2=data(:,((t3/dtm)+1):((t4/dtm)+1));

Mean2= mean(data2(2,:));

STD2=std(data2(2,:));

  

yneg=STD2;

ypos=STD2;

xneg=*e*(1,2);

xpos=*e*(1,2);

  

hold on

errorbar((t(1,2)),Mean2,yneg,ypos,xneg,xpos)

plot(data2(1,:),data2(2,:), 'r','LineWidth', 2)

  

%for third age group

t5=(t(1,3)-e(1,3));

t6=(t(1,3)+*e*(1,3));

data3=data(:,((t5/dtm)+1):((t6/dtm)+1));

Mean3= mean(data3(2,:));

STD3=std(data3(2,:));

  

yneg=STD3;

ypos=STD3;

xneg=*e*(1,3);

xpos=*e*(1,3);

  

hold on

errorbar((t(1,3)),Mean3,yneg,ypos,xneg,xpos)

plot(data3(1,:),data3(2,:), 'r','LineWidth', 2)

  

%for forth age group

t7=(t(1,4)-e(1,4));

t8=(t(1,4)+*e*(1,4));

data4=data(:,((t7/dtm)+1):((t8/dtm)+1));

Mean4= mean(data4(2,:));

STD4=std(data4(2,:));

  

yneg=STD4;

ypos=STD4;

xneg=*e*(1,4);

xpos=*e*(1,4);

  

hold on

errorbar((t(1,4)),Mean4,yneg,ypos,xneg,xpos)

plot(data4(1,:),data4(2,:), 'r','LineWidth', 2)

  

%for fifth age group

t9=(t(1,5)-e(1,5));

t10=(t(1,5)+*e*(1,5));

data5=data(:,((t9/dtm)+1):((t10/dtm)+1));

Mean5= mean(data5(2,:));

STD5=std(data5(2,:));

  

yneg=STD5;

ypos=STD5;

xneg=*e*(1,5);

xpos=*e*(1,5);

  

hold on

errorbar((t(1,5)),Mean5,yneg,ypos,xneg,xpos)

plot(data5(1,:),data5(2,:), 'r','LineWidth', 2)

  

%for sixth age group

t11=(t(1,6)-e(1,6));

t12=(t(1,6)+*e*(1,6));

data6=data(:,((t11/dtm)+1):((t12/dtm)+1));

Mean6= mean(data6(2,:));

STD6=std(data6(2,:));

  

yneg=STD6;

ypos=STD6;

xneg=*e*(1,6);

xpos=*e*(1,6);

  

hold on

errorbar((t(1,6)),Mean6,yneg,ypos,xneg,xpos)

plot(data6(1,:),data6(2,:), 'r','LineWidth', 2)

  

%for seventh age group

t13=(t(1,7)-e(1,7));

t14=(t(1,7)+*e*(1,7));

data7=data(:,((t13/dtm)+1):((t14/dtm)+1));

Mean7= mean(data7(2,:));

STD7=std(data7(2,:));

  

yneg=STD7;

ypos=STD7;

xneg=*e*(1,7);

xpos=*e*(1,7);

  

hold on

errorbar((t(1,7)),Mean7,yneg,ypos,xneg,xpos)

plot(data7(1,:),data7(2,:), 'r','LineWidth', 2)

  

%for eighth age group

t15=(t(1,8)-e(1,8));

t16=(t(1,8)+*e*(1,8));

data8=data(:,((t15/dtm)+1):((t16/dtm)+1));

Mean8= mean(data8(2,:));

STD8=std(data8(2,:));

  

yneg=STD8;

ypos=STD8;

xneg=*e*(1,8);

xpos=*e*(1,8);

  

hold on

errorbar((t(1,8)),Mean8,yneg,ypos,xneg,xpos)

plot(data8(1,:),data8(2,:), 'r','LineWidth', 2)

  

%for ninth age group

t17=(t(1,9)-e(1,9));

t18=(t(1,9)+*e*(1,9));

data9=data(:,((t17/dtm)+1):((t18/dtm)+1));

Mean9= mean(data9(2,:));

STD9=std(data9(2,:));

  

yneg=STD9;

ypos=STD9;

xneg=*e*(1,9);

xpos=*e*(1,9);

  

hold on

errorbar((t(1,9)),Mean9,yneg,ypos,xneg,xpos)

plot(data9(1,:),data9(2,:), 'r','LineWidth', 2)

  

%for tenth age group

t19=(t(1,10)-e(1,10));

t20=(t(1,10)+*e*(1,10));

data10=data(:,((t19/dtm)+1):((t20/dtm)+1));

Mean10= mean(data10(2,:));

STD10=std(data10(2,:));

  

yneg=STD10;

ypos=STD10;

xneg=*e*(1,10);

xpos=*e*(1,10);

  

hold on

errorbar((t(1,10)),Mean10,yneg,ypos,xneg,xpos)

plot(data10(1,:),data10(2,:), 'r','LineWidth', 2)

  

%for eleventh age group

t21=(t(1,11)-e(1,11));

t22=(t(1,11)+*e*(1,11));

data11=data(:,((t21/dtm)+1):((t22/dtm)+1));

Mean11= mean(data11(2,:));

STD11=std(data11(2,:));

  

yneg=STD11;

ypos=STD11;

xneg=*e*(1,11);

xpos=*e*(1,11);

  

hold on

errorbar((t(1,11)),Mean11,yneg,ypos,xneg,xpos)

plot(data11(1,:),data11(2,:), 'r','LineWidth', 2)

  

%for twelveth age group

t23=(t(1,12)-e(1,12));

t24=(t(1,12)+*e*(1,12));

data12=data(:,((t23/dtm)+1):((t24/dtm)+1));

Mean12= mean(data12(2,:));

STD12=std(data12(2,:));

  

yneg=STD12;

ypos=STD12;

xneg=*e*(1,12);

xpos=*e*(1,12);

  

hold on

errorbar((t(1,12)),Mean12,yneg,ypos,xneg,xpos)

plot(data12(1,:),data12(2,:), 'r','LineWidth', 2)

  

%for thirteenth age group

t25=(t(1,13)-e(1,13));

t26=(t(1,13)+*e*(1,13));

data13=data(:,((t25/dtm)+1):((t26/dtm)+1));

Mean13= mean(data13(2,:));

STD13=std(data13(2,:));

  

yneg=STD13;

ypos=STD13;

xneg=*e*(1,13);

xpos=*e*(1,13);

  

hold on

errorbar((t(1,13)),Mean13,yneg,ypos,xneg,xpos)

plot(data13(1,:),data13(2,:), 'r','LineWidth', 2)

%Ignore the warning massage at the end

  

%%—————-

%Save the data as matrix [mean Temp. (K) column; SD column]

All_data=[Mean13,STD13;Mean2,STD2;Mean3,STD3;Mean4,STD4;...

Mean5,STD5;Mean6,STD6;Mean7,STD7;Mean8,STD8;Mean9,STD9;...

Mean10,STD10;Mean11,STD11;Mean12,STD12;Mean1,STD1;];

## Appendix Supplementary materials

Image, application 1

Image, application 2

Image, application 3

Image, application 4
